# Diauxic growth of *Clostridium acetobutylicum* ATCC 824 when grown on mixtures of glucose and cellobiose

**DOI:** 10.1186/s13568-018-0615-2

**Published:** 2018-05-22

**Authors:** Felipe Buendia-Kandia, Emmanuel Rondags, Xavier Framboisier, Guillain Mauviel, Anthony Dufour, Emmanuel Guedon

**Affiliations:** 1 LRGP, UMR CNRS 7274, Université de Lorraine, 2 Avenue de la Forêt de Haye TSA 40602, 54518 Vandoeuvre lès Nancy, France; 2 LRGP, UMR CNRS 7274, Université de Lorraine, 1 Rue Grandville, 54000 Nancy, France

**Keywords:** Biomass, Cellulose, Cellobiose, Diauxie, Fermentation, *Clostridium acetobutylicum*

## Abstract

*Clostridium acetobutylicum*, a promising organism for biomass transformation, has the capacity to utilize a wide variety of carbon sources. During pre-treatments of (ligno) cellulose through thermic and/or enzymatic processes, complex mixtures of oligo saccharides with beta 1,4-glycosidic bonds can be produced. In this paper, the capability of *C. acetobutylicum* to ferment glucose and cellobiose, alone and in mixtures was studied. Kinetic studies indicated that a diauxic growth occurs when both glucose and cellobiose are present in the medium. In mixtures, d-glucose is the preferred substrate even if cells were pre grown with cellobiose as the substrate. After the complete consumption of glucose, the growth kinetics exhibits an adaptation time, of few hours, before to be able to use cellobiose. Because of this diauxic phenomenon, the nature of the carbon source deriving from a cellulose hydrolysis pre-treatment could strongly influence the kinetic performances of a fermentation process with *C. acetobutylicum.*

## Introduction

Lignocellulosic biomass represents an interesting alternative to fossil carbon resources (McKendry 2002; Mosier et al. 2005; Wyman et al. 2005; Briens et al. 2008; Wettstein et al. 2012; Nanda et al. 2014).

Indeed, lignocellulosic biomass can be transformed into energy and fuels through a variety of chemical, thermo-chemical and biological conversion processes. Among biological conversion processes, anaerobic digestion and more specifically fermentation processes are the most implemented in industries (Lin and Tanaka 2005; Azman et al. 2015), because it offers the most important biological conversion ways to transform a wide variety of organic materials mainly coming from agroindustry such as by-products into fuels and chemical products with higher values (McKendry 2002; Briens et al. 2008, Turon et al. 2016). However, fermentation processes need an efficient monitoring and control to ensure optimal conditions for productions and yields (Lin and Tanaka 2005; Azman et al. 2015).

Bioethanol, one of the main fermentation products, is predominantly produced using yeast through the fermentation of easily degradable carbohydrate substrates, such as corn starch and sugar cane (Henstra et al. 2007). Nonetheless, the utilization of these kinds of substrates to produce biofuels threatens food supplies and biodiversity. For this reason, the production of new generation biofuels using bacteria may offer an interesting alternative to this problem (Ranjan and Moholkar 2012; Morone and Pandey 2014). As an alternative, some *Clostridium* species can produce acetone–butanol–ethanol (ABE) from renewable resources, such as biomass and derivatives (Lee et al. 2008; Tracy et al. 2012; Jang et al. 2012; Gu et al. 2014). Butanol can be blended with gasoline or used directly as a fuel for transportation (Qureshi and Ezeji 2008; Pfromm et al. 2010; Ranjan and Moholkar 2012). For this purpose, a diversity of *Clostridium* strains coming from the butyric and butylic groups has been studied (*C. saccharoperbutylacetonicum, C. acetobutylicum, C. beijerinckii, C. butyricum*) (Lee et al. 2008; Jang et al. 2012; Gu et al. 2014).

*C. acetobutylicum* is one of the most studied species thanks to its capability of producing butanol and hydrogen by anaerobic fermentation in high yields, from a wide range of substrates (Qureshi and Ezeji 2008; Survase et al. 2011b; Napoli et al. 2011; Li et al. 2012; Jurgens et al. 2012; Gao et al. 2014; Aristilde et al. 2015; Raganati et al. 2015). This strain was largely used in the ABE fermentation process during the 1910s until the end of 1950s, because the petrochemical industry development led to the decline of this fermentation as an industrial process (Jones and Woods 1986). An important feature of the ABE fermentation is its biphasic development leading to two distinct groups of metabolic products. The first phase is the acidogenesis, which is characteristized by acids (mainly butyric, acetic and lactic acid) and hydrogen production. During acidogenesis, the cells will usually display an active growth including an exponential growth phase (Andersch et al. 1983; Hartmanis and Gatenbeck 1984). The second phase is the solventogenesis, which is characteristized by organic acids re-assimilation and solvent production, with butanol, acetone and ethanol as the major products (Monot et al. 1984). The transition between the two phases is the result of strong gene expression changes (Grupe and Gottschalk 1992; Girbal et al. 1995).Nowadays, with the depletion of oil, renewable processes to replace fossil carbon need to be developed. But, a major factor that determines the viability of this process is the cost of feedstock. In this regard, lignocellulosic biomasses are considered as interesting feedstocks for fermentation (Ezeji et al. 2007; Jang et al. 2012), Whereas its complex structure composed of cellulose embedded in a complex hemicellulose and lignin matrix may hinder biological conversion due to a high resistance to most chemical and biological pretreatments (Wyman et al. 2005; Kumar et al. 2009; Rinaldi and Schüth 2009; Hendriks and Zeeman 2009; Alvira et al. 2010; Jurgens et al. 2012).

To enhance the fermentability of cellulose a preliminary treatment is frequently used. Chemical, thermal and enzymatic pre-treatments allow to produce a mixture of carbohydrates and other organic compounds that are soluble and accessible to bacteria (Kumar et al. 2009; Rinaldi and Schüth 2009; Alvira et al. 2010; Ibrahim et al. 2015). The conversion of each one of these compounds into added-value products and building blocks is crucial to increase the efficiency of an integrated bio-refinery, and the understanding the metabolism of individual carbohydrates and mixtures is very important to lower the cost of fermentation process. This and the ability of saccharolytic clostridia to use a wide range of carbohydrates have prompted research dedicated to the production of a cheaper substrates (Jones and Woods 1986; Lee et al. 2008; Tracy et al. 2012; Gu et al. 2014).

In addition, the growth of *C. acetobutylicum* on a mixture of substrates has been already studied using different combinations of carbon sources (glucose and xylose, glucose and mannose, d-glucose and glycerol, …) and different metabolic responses were obtained according to the nutritional environment (Mes-Hartree and Saddler 1982; Ounine et al. 1985; Fond et al. 1986; Vasconcelos et al. 1994; Mitchell et al. 1995; Survase et al. 2011a). Recently, the interest in *C. acetobutylicum* as un effective biofuel producer of butanol instead of ethanol from lignocellulose derivated substrates (glucose, cellobiose and xylose) has increased, resulting in numerous studies privileging mainly butanol production from xylose alone or in mixture with glucose rather than with cellobiose (Patakova et al. 2013; Nogué and Karhumaa 2015; Raganati et al. 2015; Zhao et al. 2016) whereas this last substrate with glucose and other cello-oligosaccharides in mixture would be also expected to be major products of lignocellulose degradation.

Indeed, despite a recent proteomic study describing the influence of lignin in the metabolic behavior of *C. acetobutylicum* ATCC 824 with cellobiose as the substrate (Raut et al. 2016), the capability of *Clostridium* strains to ferment mixtures of cellobiose and glucose in synthetic medium is not well studied yet. Furthermore, bacteria have developed mechanisms that allow them to use selectively mixtures of different carbon sources (Mitchell et al. 1995).

In this study, kinetics of *C. acetobutylicum* cultivated with glucose and/or cellobiose, two substrate models representatives of cellulose hydrolysis products were compared. Only the acidogenic phase in which the active growth phase and substrate consumptions could be studied was considered. Transitions and re-assimilation of acids mechanisms were excluded from this work.

## Materials and methods

### Microorganism and media

Spores of *Clostridium acetobutylicum* ATCC 824 were maintained in Difco™ Reinforced Clostridial Medium (RCM) at ambient temperature. Whereas precultures were different for each culture experiment, they were prepared rigorously in a similar way. Each experiment was started with the spores of *C. acetobutylicum*. The spore culture was diluted to a concentration of 10% in 10 mL of RCM fresh media (Hungate tubes) and then heat shocked at 80 °C for 20 min to induce germination. Reactivated cultures were incubated in fresh RCM medium at 37 °C for 12 h and then transferred into 30 mL (pre-culture tubes) of a synthetic medium. In fact, the volume of preculture was prepared depending of the final volume of culture in order to get a ratio of 1/4. The synthetic medium was composed of 20 g/L glucose, 0.5 g/L KH_2_PO_4_, 1.5 g/L (NH_4_)_2_SO_4_, 1 g/L MgCl_2_, 0.15 g/L CaCl_2_, 1.5 g/L yeast extract, 0.01 g/L FeSO_4_.7H_2_O, 0.01 g/L MnSO_4_.H_2_O, 3 g/L CaCO_3_, 4 × 10^−5^ g/L biotin. All the Chemicals, yeast extract and biotin were provided by Sigma Aldrich.

### Fermentation

Batch fermentations were carried out in a bioreactor controlled by an Applikon ADI 1030 bio controller (Applikon Biotechnology). Throughout all fermentation experiments, temperature was maintained at 37 °C and pH at 5.5 by the automatic addition of 3 N NaOH, 1 N HCl using the biocontroler and a pH probe (Mettler Toledo). The bioreactor was initially purged with nitrogen to ensure an anaerobic atmosphere and then inoculated with 6% (v/v) active growing pre-cultures into 1.5 L of synthetic media with the same composition of the pre-culture media (without CaCO_3_ to prevent interference with DO measurements). The glucose concentrations for the fermentation studies were comprised between 25 and 35 g/L (138–195 mM), unless otherwise indicated. Other experiments with cellobiose were carried out with the same glucose-equivalent mass concentration. However, controlling residual substrate from pre-culture was not possible. For this reason, the initial substrate concentration was not exactly the same. These variations did not alter the study case and the desired results. Each experience was carried out until complete substrate consumption. All the experiments were performed at least in duplicate.

### Analyses

Cell density was measured at 600 nm using a spectrophotometer (HITACHI U-2000). The relationship between the cell dry weight and the optical density was established thanks to a calibration made by triplicate using spectrophotometer at 600 nm. The correlation factor found was 0.346 g/L cell dry weight per unity of absorbance. This value was in agreement with others related in the literature (Kim et al. 1984).

Glucose, cellobiose, acetone, ethanol, butanol, acetic acid, lactic acid and butyric acid concentrations were measured by high-performance liquid chromatography equipped with a refractive index detector and an ultraviolet–visible spectroscopy (HPLC-RID-UV) using a Aminex HPX 87 h column. The samples were filtered with a 0.2 µm filter and the injection volume was 10 µL. The oven kept the column at 45 C; the mobile phase was a 25 mM sulfuric acid (H_2_SO_4_) solution. The analysis time was 35 min in isocratic mode.

### Calculations

The main products of glucose or cellobiose fermentation by *C. cellulolyticum* were acetate, Butyrate, ethanol, butanol, acetone, lactate, H_2_, and CO_2_, as previously described (Vasconcelos et al. 1994) Carbon recoveries were calculated from the production of metabolites, and biomass, present in the supernatant. Biomass was taken into account on the basis of the cell dry weight and a mean biomass formula of C_4_H_7_O_2_N (Guedon et al. 1999). According to the metabolic scheme (Vasconcelos et al. 1994), the conversion of glucose to products can be written as follows:

$${\text{glucose}}\, + \, 2\,{\text{ADP}}\, + \, 2\,{\text{NADH}}\, \to \, 2\,{\text{ethanol}}\, + \, 2\,{\text{ATP}}\, + \, 2\,{\text{NAD}}^{ + } \, + \, 2\,{\text{CO}}_{ 2} \, + \, 2\,{\text{H}}_{ 2}$$

$${\text{glucose}}\, + \, 4\,{\text{ADP}}\, + \, 2\,{\text{NAD}}^{ + } \, \to \, 2\,{\text{acetate}}\, + \, 4\,{\text{ATP}}\, + \, 2\,{\text{NADH}}\, + \, 2\,{\text{CO}}_{ 2} \, + \, 2\,{\text{H}}_{ 2}$$

$${\text{glucose}}\, + \, 3\,{\text{ADP}}\,\, \to \,{\text{butyrate}}\, + \, 3\,{\text{ATP}}\, + \, 2\,{\text{NADH}}\, + \, 2\,{\text{CO}}_{ 2} \, + \, 2\,{\text{H}}_{ 2}$$

$${\text{glucose}}\, + \, 2\,{\text{ADP}}\, + \, 2\,{\text{NADH}}\, \to \,{\text{butanol}}\, + \, 2\,{\text{ATP}}\, + \, 2\,{\text{ NAD}}^{ + } \,\, + \, 2\,{\text{CO}}_{ 2} \, + \, 2\,{\text{H}}_{ 2}$$

$${\text{glucose}}\, + \, 2\,{\text{ADP}}\, + \, 2\,{\text{NAD}}^{ + } \, \to \,{\text{acetone}}\, + \, 3\,{\text{ATP}}\, + \, 2\,{\text{NADH}}\, + \, 3\,{\text{CO}}_{ 2} \, + \, 2\,{\text{H}}_{ 2}$$

For example, from the last stoichiometric equation, each acetone molecule produced is associated to the formation of 3 CO_2_ molecules. Therefore, the CO_2_ production was calculated on the basis of product formation, as the sum of [acetate], 2*[butyrate], [ethanol], 2*[butanol], and 3*[acetone] concentrations.

CO_2_ production was calculated as the sum of [acetate], 2*[butyrate], [ethanol], 2*[butanol], and 3*[acetone] concentrations.

## Results

### *General growth and metabolic features of* Clostridium acetobutylicum *ATCC 824 cultivated with glucose or cellobiose*

**Fig. 1:**
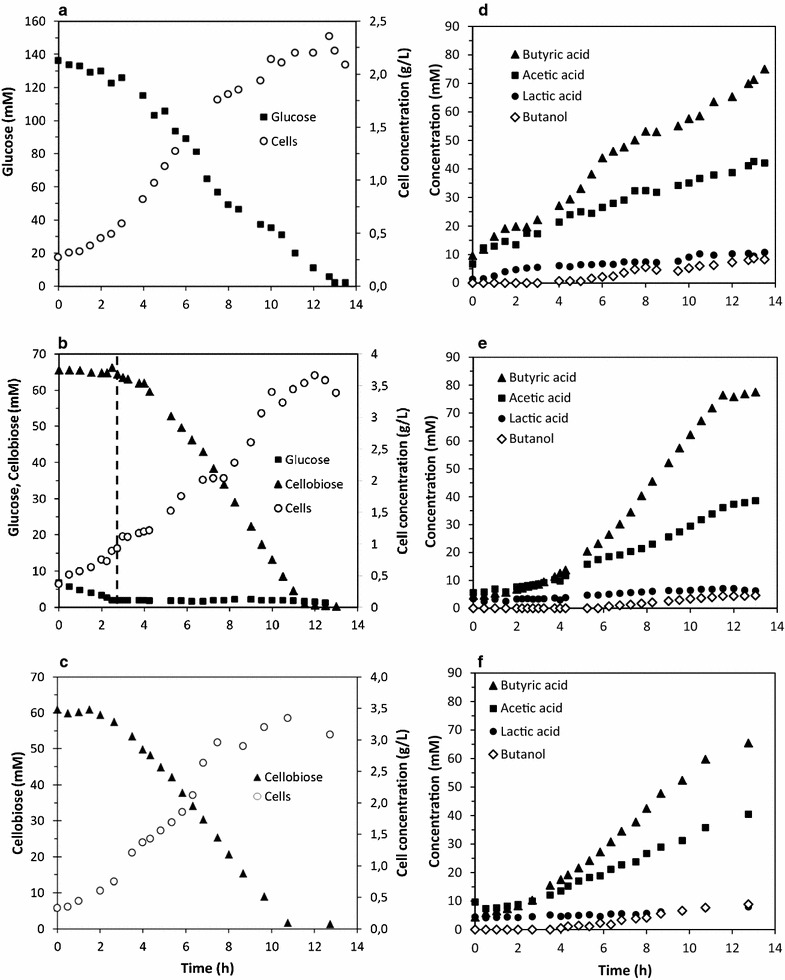
Kinetics of growth and metabolism of glucose or cellobiose as the substrate by *C. acetobutylicum*. **a** Glucose as the substrate (136 mM) and glucose-pregrown cells as the inoculum. **b** Cellobiose as the substrate (67 mM) and glucose-pregrown cells as the inoculum. The dashed line represents le beginning of the lag phase after glucose was exhausted. (c) Cellobiose as the substrate (67 mM) and cellobiose-pregrown cells as the inoculum. **d**–**f** are the respective product kinetics of (**a**–**c**). Each graph is representative of at least three independent experiments (n = 3)

Kinetics of growth and metabolism of *C. acetobutylicum* with glucose or cellobiose as the carbon and energy source were performed with precultures carried out with either glucose or cellobiose (Fig. 1). Pre cultures and cultures performed with the same carbon source were compared with precultures and cultures performed with the switched substrates. The pH was set at 5.5 in order to favor acidogenesis, resulting In butyric, acetic and lactic acids, as the main products whatever the substrate used.

The carbon balances for these experiments were comprised between 90 and 98%, indicating that most of the carbon substrates and products have been taken into account. Therefore, resulting kinetics can be fully interpreted.

Figure 1a shows the growth kinetic of *C. acetobutylicum* on glucose inoculated with a preculture grown with the same substrate. Taking into account the residual substrate coming from the preculture, the initial glucose concentration was 136 mM (25 g/L). After approximately 13 h of culture, all the glucose was completely consumed and no lag phase was observed since the culture rapidly entered in exponential phase. In these experimental conditions, a maximal specific growth rate (µ_max_) of 0.26 h^−1^ was reached after 3 h of culture whereas a maximal cell concentration of 2.36 g/L (dry cell weight) was obtained when glucose was entirely depleted. Glucose fermentation by *C. acetobutylicum* was accompanied by the accumulation of acidic products (Fig. 1d) such as butyric, acetic and lactic acid up to 74, 40 and 9 mM respectively, whereas butanol was a minor product with a production of only 8 mM.

Figure 1b and c report the growth kinetics of *C. acetobutylicum* with cellobiose (67 and 61 mM respectively) as the carbon and energy source, and with glucose or cellobiose as the respective preculture substrates.

When inoculated with a preculture cultivated with glucose, an immediate and short growth phase occurred during the 3 first hours of culture (Fig. 1b). During this phase, no cellobiose was used, whereas glucose coming from preculture (7.2 mM) was completely exhausted. Then, the consumption of cellobiose by *C. acetobutylicum* occurred until exhaustion and was associated with an active cell growth.

Interestingly, as indicated by the dashed line (Fig. 1b), the use of cellobiose was accompanied by a short lag phase during approximately 2 h after glucose exhaustion. Then the growth of *C. acetobutylicum* started and reached a µ_max_ of 0.21 h^−1^ and maximal biomass concentration of 3.7 g/L after 12 h of culture.

As observed during fermentation kinetics performed with glucose in Fig. 1a, the product pattern obtained with cellobiose as the carbon source and with glucose as the preculture substrate (Fig. 1e) is similar since butyric (76 mM), acetic (35 mM) and lactic acids (5.5 mM) were the main products at the end of the culture whereas butanol accumulated at a low concentration.

Figure 1c displays the growth kinetic of *C. acetobutylicum* with cellobiose as the substrate (61 mM) after an inoculation with a preculture grown with cellobiose. In less than 11 h, all the cellobiose was fully consumed by *C. acetobutylicum*, resulting in an active cell growth without apparent lag phase. In these conditions, a µ_max_ of 0.23 h^−1^ and a maximal cell concentration of 3.4 g/L after 8 h of culture were observed. Similarly to the previous kinetics (Fig. 1a, b), the product pattern obtained with cellobiose as the carbon source in the fermentation broth and in precultures (Fig. 1f) are similar (Fig. 1d, e), indicating that neither the carbon source in precultures, nor the carbon source in cultures have an influence on the carbon distribution during fermentation.

However, when pre-cultures were grown with glucose as the substrate (Fig. 1b), an adaptation stage of *C. acetobutylicum* seems to be important before to be able to use cellobiose as the main carbon and energy source.

### Fermentation of glucose and cellobiose mixtures by *C. acetobutylicum*

In order to get a better understanding of the lag phase observed during the cellobiose culture inoculated with pre-cultures performed with glucose (Fig. 1b), experiments using mixtures of glucose and cellobiose were performed with either glucose and cellobiose pre-cultures as the inoculum. These experiments were carried out in similar conditions to the previous ones. Glucose and cellobiose were entirely consumed and typical acidogenesis product profiles were observed in batch fermentations. In these experimental conditions, the carbon recoveries were a bit lower than previously and comprised between 85 and 87%.

**Fig. 2:**
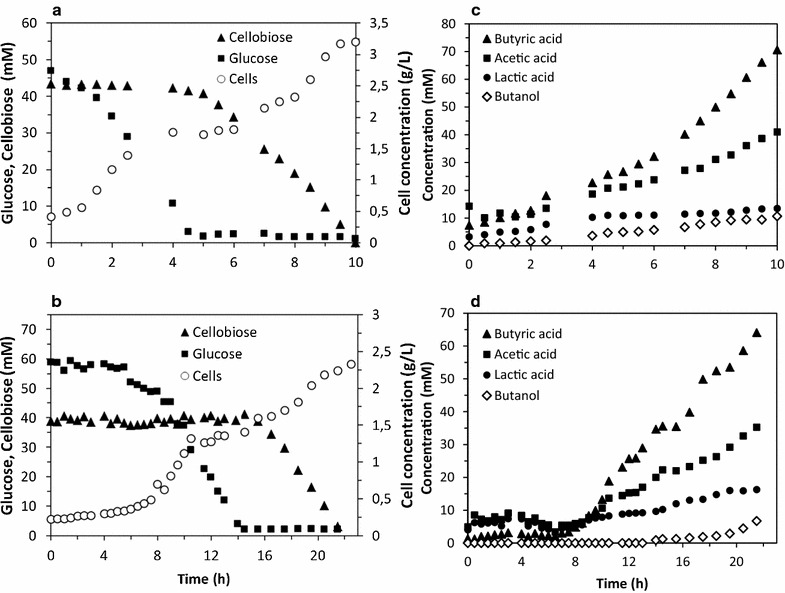
Kinetics of growth and metabolism of d-glucose/cellobiose mixtures by *C. acetobutylicum*. **a** glucose-pregrown cells as the inoculum, initial glucose or cellobiose concentrations were 46.97 and 43.30 mM respectively. **b** cellobiose-pregrown cells as the inoculum, initial glucose or cellobiose concentrations were 58.7.97 and 38.8 mM respectively. **c**, **d** are the respective product kinetics of (**a**, **b**). Each graph is representative of at least three independent experiments (n = 3)

Figure 2a reports sequential consumption of glucose and cellobiose as well as growth kinetics observed when pre-cultures were performed with glucose. In this experiment, the culture medium contained a glucose and cellobiose mixture with respective initial concentrations of 47 and 43.3 mM.

During this fermentation process, no lag phase was observed and glucose was the first substrate to be consumed by cells. In these experimental conditions, a µ_max_ of 0.28 h^−1^ was measured; resulting in a cell concentration of 2 g/L. Cellobiose began to be consumed by *C. acetobutylicum* in a second time and only when glucose was completely exhausted after 4.5 h of culture. During the first 2 h of cellobiose utilization, a temporary growth cessation was observed. Then, cellobiose was fully consumed, in association with a second growth, resulting in a maximal cell concentration of 3.2 g/L, and a µ_max_ of 0.22 h^−1^. However and as initially observed in Fig. 1b, a lag phase was observed when glucose was exhausted suggesting that an adaptation phase may be required before cellobiose utilization *by C. acetobutylicum*.

Figure 2b reports sequential consumption of glucose and cellobiose as well as growth kinetics observed when the fermentation process was inoculated with cellobiose pregrown cells. The respective initial concentrations of glucose and cellobiose in the bioreactor were 58.7 and 38.8 mM. Interestingly, a lag phase of 4 h without any glucose or cellobiose consumption was observed. Then cells started to use glucose until exhaustion. Then cellobiose utilization started about 2 h after glucose was entirely depleted. However, the maximal growth rate was barely slower with cellobiose (µ_max_ of 0.22 h^−1^), compared to glucose (0.27 h^−1^). Product patterns (Fig. 2c, d) obtained with mixtures of glucose and cellobiose were similar to those previously observed in Fig. 1e–g whatever the culture conditions.

In this study, results strongly suggest that glucose is definitely the preferred substrate, since it is always used in first place, even if pre-cultures were performed with cellobiose are exposed to a mixture of glucose and cellobiose. However, glucose consumption does not start immediately a lag phase is always observed when carbon substrates in pre cultures and in cultures are different, suggesting that a physiological adaptation may occur, resulting in a growth cessation. In fact, glucose and cellobiose are able to influence the consumption of each other by *C. acetobutylicum*.

## Discussion

In this study, fermentations of glucose and/or cellobiose by *C. acetobutylicum* were performed in batch mode. Mono-substrate cultures showed that *C. acetobutylicum* is able to grow with cellobiose as efficiently as with glucose.

With glucose as the substrate, kinetics of growth, substrate consumption and products formation were in good agreement with previous studies (Jones and Woods 1986; Girbal et al. 1995). Interestingly, no major difference was observed when cellobiose was the sole carbon substrate: specific growth rates measured with both substrate were almost similar, and no significant change in fermentation patterns (acidogenic phase) was observed. In fact, little was known about the cellobiose metabolism by *C. acetobutylicum* and such a study was never deeply investigated so far. Indeed, only studies dedicated to enzymatic activities expressed by *C. acetobutylicum* during the fermentation of cellobiose were reported in the literature (Allcock and Woods 1981; Mes-Hartree and Saddler 1982; Lee et al. 1985; López-Contreras et al. 2000.

However, and contrary to C*. thermocellum* (Weimer and Zeikus 1977), cultures grown on glucose/cellobiose mixtures demonstrated that *C. acetobutylicum* was unable to co-utilize both substrates at the same time and glucose was consistently the preferred carbon source. In the present work, growth cessations were always observed after glucose exhaustion and before cellobiose utilization, resulting in a second growth phase.

This phenomenon was reported for the first time by (Monod 1942), and was called “diauxie”. It is nowadays better known under the generic term of catabolic repression since some substrates have the ability to repress the expression of genes encoding catabolic enzymes and/or protein transporters, as already described in bacterial species (Magasanik 1961; Brückner and Titgemeyer 2002; Deutscher et al. 2006; Deutscher 2008; Görke and Stülke 2008) but these mechanisms are not the same for each strain and their complete characterization is still being studied.

Besides, the effect of carbon source on cell growth and fermentation products by *C. acetobutylicum* has been previously studied for different mixtures (Vasconcelos et al. 1994). In continuous cultures, *C. acetobutylicum* grown with glucose at neutral pH, produced only acids. In the same conditions, but with substrate mixtures of glucose and glycerol, alcohols were produced in higher yields but no catabolic repression was observed with these carbon sources. However, the repression of lactose transport system by glucose (Yu et al. 2007) as well as diauxic growths of *C. acetobutylicum* cultivated on mixtures of xylose and glucose (Jiang et al. 2014) have been reported, demonstrating that these mechanisms are of a great importance and participate to the control of carbon catabolic fluxes of cells depending on the nature of carbon sources.

In this study, similar mechanisms could be involved according to our results. Indeed, two putative PTSs (phosphotransferase systems) operon genes, strongly induced by cellobiose with functions connected to cellobiose metabolism, were reported by Servinsky et al. (2010), suggesting that *C. acetobutylicum* displays multiple mechanisms to import, phosphorylate and hydrolyse B-glucosides for entry into glycolyse. Besides, the similarity of the *C. acetobutylicum* PTS to PTSs found in other well characterized low GC gram positive bacteria, has led to the suggestion that they may also play a role in carbon catabolite repression (Behrens et al. 2001; Saier and Reizer 1992; Singh et al. 2008; Tangney et al. 2003).This hypothesis was reinforced by the fact that a recent proteomic study revealed that such PTS operons, especially those involved in cellobiose uptake in *C. acetobutylicum* was shown to be down regulated in the presence of lignin residues, in mixture with cellobiose (Raut et al. 2016). In this study, our results suggests that an analogous phenomenon may exist that was never reported for *C. acetobutylicum* when cultivated with mixtures of glucose and cellobiose, and therefore is an original contribution to the comprehension of carbohydrate metabolism by *Clostridial* sp.

Besides, diauxic growths observed during the kinetic studies may have a great importance for ABE fermentation processes of pretreated (ligno) cellulosic biomass. From a technological point of view, this observation may be of critical importance if industrial substrates mixes were to be used for hydrogen and/or other chemical precursor productions by *C. acetobutylicum*, since substrate consumption discontinuities could occur and may affect production performances and productivities of processes.

More studies with other complex mixtures of cellulosic derivatives as well as with various operating conditions will help to understand how *C. acetobutylicum* can manage its carbohydrate metabolism in order to perform an efficient ABE production process.

## Abbreviations

ATCC: American Type Culture Collection
ABE: acetone–butanol–ethanol
PTS: phosphotransferase system
RCM: Reinforced Clostridial Medium
